# Effects of high and low 17β-estradiol doses on focal cerebral ischemia in rats

**DOI:** 10.1038/srep20228

**Published:** 2016-02-03

**Authors:** Edvin Ingberg, Elvar Theodorsson, Annette Theodorsson, Jakob O. Ström

**Affiliations:** 1 Department of Clinical Chemistry and Department of Clinical and Experimental Medicine, Linköping University, Linköping, Sweden; 2 Division of Neuroscience, Department of Clinical and Experimental Medicine, Faculty of Health Sciences, Linköping University, Department of Neurosurgery, Anaesthetics, Operations and Specialty Surgery Center, Region Östergötland; 3 Vårdvetenskapligt Forskningscentrum/Centre for Health Sciences, Örebro University Hospital, Region Örebro Län, Örebro, Sweden; 4 School of Health and Medical Sciences, Örebro University, Örebro, Sweden

## Abstract

The majority of the numerous animal studies of the effects of estrogens on cerebral ischemia have reported neuroprotective results, but a few have shown increased damage. Differences in hormone administration methods, resulting in highly different 17β-estradiol levels, may explain the discrepancies in previously reported effects. The objective of the present study was to test the hypothesis that it is the delivered dose *per se*, and not the route and method of administration, that determines the effect, and that high doses are damaging while lower doses are protective. One hundred and twenty ovariectomized female Wistar rats (n = 40 per group) were randomized into three groups, subcutaneously administered different doses of 17β-estradiol and subjected to transient middle cerebral artery occlusion. The modified sticky tape test was performed after 24 h and the rats were subsequently sacrificed for infarct size measurements. In contrast to our hypothesis, a significant negative correlation between 17β-estradiol dose and infarct size was found (p = 0.018). Thus, no support was found for the hypothesis that 17β-estradiol can be both neuroprotective and neurotoxic merely depending on dose. In fact, on the contrary, the findings indicate that the higher the dose of 17β-estradiol, the smaller the infarct.

Stroke constitutes a substantial burden for individuals and the society, both regarding suffering and economy^1^. Despite major scientific efforts and advancing understanding of the condition, the therapeutic options are still few^2^,^3^. The search for new treatments, as well as further investigation into what factors affect the risk of having a stroke, continues.

The fact that premenopausal women suffer less from stroke than men of the same age, and that the risk increases postmenopausally, led to the hypothesis that estrogens could be neuroprotective^4^ and initiated intense research activity. Many animal studies on the effects of estrogens on cerebral ischemia, induced by middle cerebral artery occlusion (MCAo), have been performed and although the majority have reported neuroprotective results^5^,^6^, some have also shown increased damage^7^,^8^, and a full explanation still remains elusive.

One of the rat studies showing increased stroke damage after estrogen treatment was performed in our lab^9^. This finding was unexpected and prompted us to thoroughly investigate the methodological aspects, particularly the mode of hormone administration. The most commonly used administration methods for 17β-estradiol to rodents were examined in several experiments and it was shown that the commercial slow-release pellets (from the company Innovative Research of America) that we had used, produced very high hormone concentrations both in rats and mice^10^,^11^,^12^. Also, by meta-analyzing all studies on the effects of estrogens of cerebral ischemia in rats it was found that the slow-release pellets had been used in all studies showing detrimental effects, and that pellets rendered estrogens significantly more damaging/less protective than if the estrogens were administered via injections or silastic capsules^13^,^14^. To test this experimentally, the previous rat stroke study was repeated, but now 17β-estradiol was delivered in silastic capsules instead of pellets. Interestingly, we now found a protective effect, corroborating the results from the meta-analysis. However, it remained to be proven that estrogens both exert neuroprotection and promote detrimental actions depending merely on dose and independent of the administration route. An attempt at this was made in our laboratory previously but the results were inconclusive due to large random variation in outcome^15^.

The objective of the current study was therefore to test, with improved experimental setup and substantially increased group sizes, whether a high-dose silastic capsule (designed to give rise to similar concentrations of estrogens as the neurotoxic pellets) would increase ischemic damage, whereas a low-dose capsule would decrease the damage.

## Results

### Infarct sizes, modified sticky tape test and mortality

**Figure 1:**
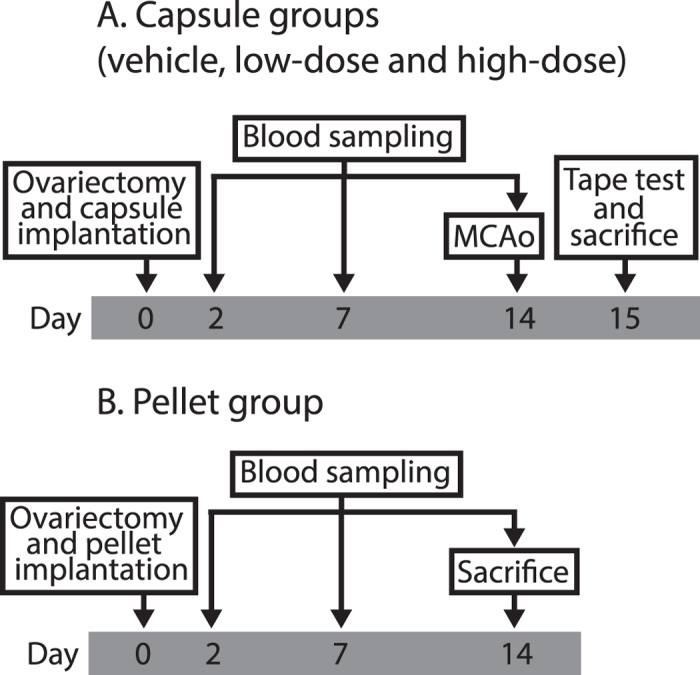
Overview of the experiment. All groups were ovariectomized and administered 17β-estradiol or vehicle on day 0. After blood samplings on day 2, 7 and 14 the capsule groups (n = 40 per group) were subjected to middle cerebral artery occlusion (MCAo) on day 14 whereas the pellet group (n = 10) was sacrificed. After the modified sticky tape test (MST) on day 15, the capsule groups were also sacrificed.

**Figure 2:**
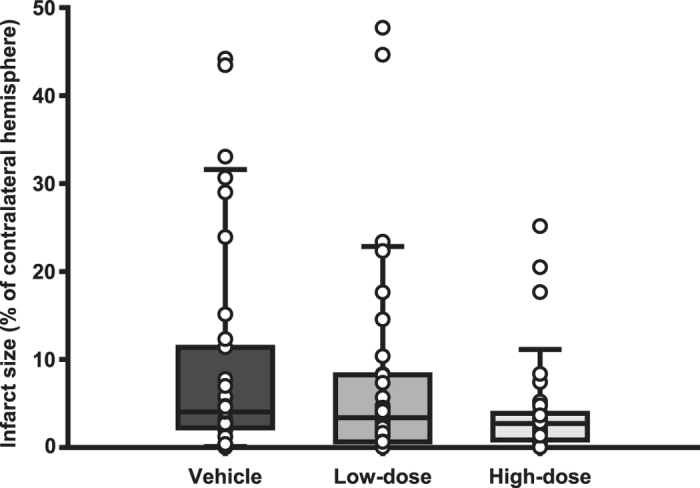
Infarct sizes of groups subjected to middle cerebral artery occlusion (MCAo). No significant differences were found between the groups (p = 0.054). However, a trend towards more favorable infarct outcome with higher 17β-estradiol dose was noted, which was confirmed by Spearman correlation analysis (p = 0.018). Whiskers indicate the 90^th^ and 10^th^ percentiles.

**Figure 3:**
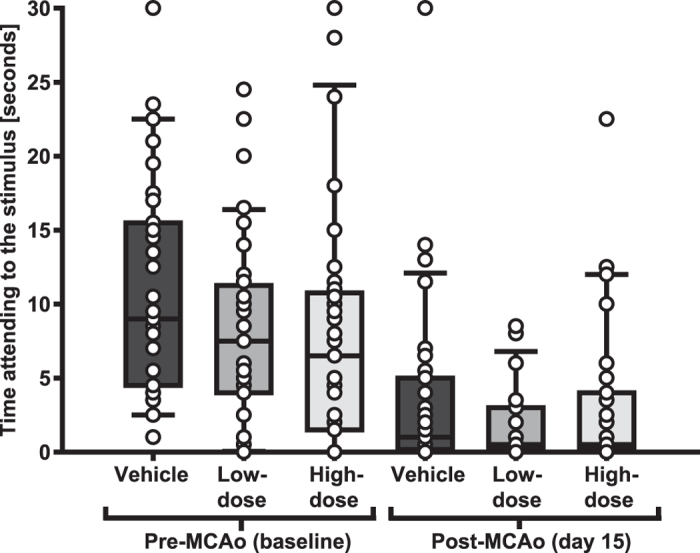
Modified sticky tape test (MST). Time attending to the stimulus, i.e. time spent trying to remove the tape sleeve from the right forepaw was recorded. No significant differences were found between the groups (p = 0.705). Whiskers indicate the 90^th^ and 10^th^ percentiles.

One hundred and twenty rats were randomized into three groups that were ovariectomized and via subcutaneous silastic capsules administered vehicle (vehicle group; n = 40), low-dose 17β-estradiol capsules (low-dose group; n = 40) or high-dose 17β-estradiol capsules (high-dose group; n = 40) on day 0. After two weeks (day 14) the rats were subjected to middle cerebral artery occlusion (MCAo) and on day 15 the modified sticky-tape test was performed and subsequently the rats were sacrificed for lesion measurement (for an overview of the experiment see Fig. 1). Total mortality post-MCAo (included mortality; Table 1) was 5%. In the non-parametric analyses of infarct size and MST outcome with this mortality included as worst possible outcome, no significant differences were found between the MCAo groups (p = 0.054 and p = 0.705, respectively; Figs 2 and 3). Since this total outcome model (mortality included) is a novel statistical approach, Welch’s ANOVA (due to unequal variances) and one-way ANOVA with the effect of group on infarct size and MST outcome, respectively, were performed to confirm and these did not show any significant differences between the groups either (p = 0.052 and p = 0.316, respectively). In both the non-parametric and the parametric analyses, the p-values for infarct size were just above the significance level and the trend was towards more protection with higher dose; the infarct sizes in the high-dose group were close to being significantly smaller than those in the vehicle group in the post-hoc analysis (p = 0.055). Further, when plotting infarct sizes, it was clear that a trend towards a more favorable outcome with higher 17β-estradiol dose existed (Fig. 2). A Spearman correlation between 17β-estradiol dose (group) and infarct outcome (non-parametric, with mortality as worst outcome as above) was therefore performed in addition to the statistical analyses decided on *a priori*. A significant negative correlation was found (p = 0.018), meaning that the higher the treatment dose, the smaller the infarcts of that group.

No rats were excluded based on the degree of cerebral perfusion decrease (LDF decrease). Only seven animals had a decrease of less than 50% (average decrease 66 ± 1%) at filament insertion compared to baseline and no correlation was seen between LDF decrease and infarct size (r = 0.09).

### Serum 17β-estradiol concentrations

Blood samples were drawn from the rats administered 17β-estradiol via silastic capsules on days 2, 7 and 14 (10 rats per group). In addition, another group of rats (pellet group; n = 10) were ovariectomized and administered high-dose 17β-estradiol slow-release pellets (day 0) and blood sampled (days 2, 7 and 14) to enable comparison of obtained 17β-estradiol levels between the capsules and the pellets (see Fig. 1). The high-dose group had significantly higher levels than the low-dose group on all sampling days (p = 0.0001, p = 0.03 and p = 0.01 for days 2, 7 and 14, respectively) and higher than the vehicle group on days 2 and 14 (p < 0.0001 and p = 0.03) but not day 7. The pellet group had higher concentrations than the vehicle group on all occasions (p = 0.005, p < 0.0001 and p < 0.0001 for days 2, 7 and 14, respectively) and higher than the low-dose group on days 7 and 14 (p < 0.0001 and p = 0.01) but not day 2. No significant differences in concentrations were seen between the high-dose and the pellet groups or between the low-dose and vehicle groups on any of the blood sample days. Concentrations are presented in Table 2.

### Body weights

**Figure 4:**
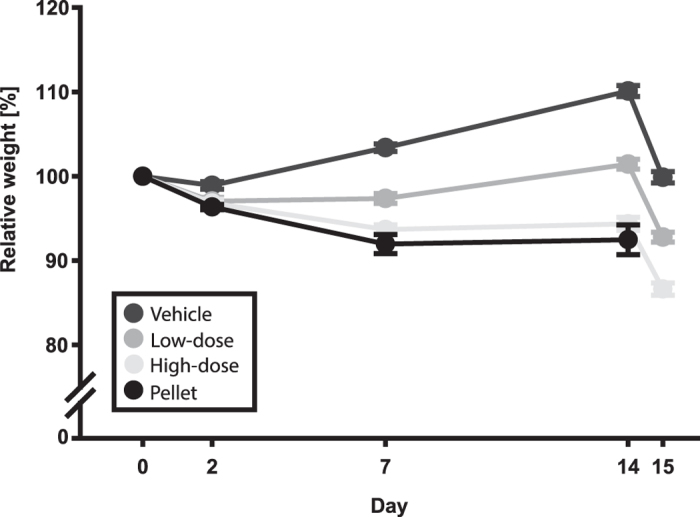
Body weight profiles. Weights were normalized to each animal’s value pre-ovariectomy. Over time, body weights of the pellet group was significantly lower than both the vehicle (<0.001) and low-dose (p = 0.008) groups whereas the high-dose group was significantly lower than the vehicle group (p < 0.001). Mean ± SEM.

As depicted in Fig. 4, weights in the different groups diverged after ovariectomy until MCAo with the vehicle group increasing over time, the low-dose being relatively stable and the high-dose and pellet groups decreasing. A main effect of group on weight, with measuring days 2, 7 and 14 included in the analysis (post-ovariectomy up until pre-MCAo), was found (p < 0.001). In the subsequent post-hoc pairwise comparisons, significant differences were found between the vehicle and high-dose groups (p < 0.001), the vehicle and pellet groups (p < 0.001) and between the low-dose and pellet groups (p = 0.008).

### Protocol violations

Seven rats died before or during MCAo (excluded mortality, see Exclusion criteria below) and these were excluded according to the exclusion criteria. One of the surviving rats did not contribute with infarct size due to technical problems during scanning of brain slices. Four samples had 17β-estradiol levels above the measurement range of the assay despite dilution, the concentrations in these cases were set to the upper limit of the measurement range.

## Discussion

No statistically significant differences were found between the groups in the main analyses regarding infarct size or behavioral outcome. Thus, the hypothesis that the high-dose group would have the most severe damage while the low-dose group would have the mildest was not confirmed. In contrast to the hypothesis, a significant negative correlation was found between 17β-estradiol dose group and infarct size meaning that the higher the dose, the smaller the infarcts.

In the meta-analysis from 2009, mentioned above, it was proposed that mode of estrogen administration resulting in different hormone concentrations might account for the discrepancy of the previous literature regarding estrogens effect on ischemic stroke^13^ and this formed the basis for the present study. A recent update of that meta-analysis corroborated the idea; the slow-release pellets, proven to on some occasions produce very high levels of 17β-estradiol, were more prone to render estrogens damaging compared to injection and silastic capsule treatment^14^. Further, we showed in the first of these two meta-analyses that higher pellet dose was associated to a neurotoxicity^13^. The question of dose has also been addressed experimentally in a study by Ma and collegues^16^. In that study, low-dose 17β-estradiol was demonstrated to be protective both *in vitro* and *in vivo*; however, regarding the high-dose treatment, attenuated protection rather than augmented damage was seen *in vivo* whereas actual toxic effects were found *in vitro*. In the light of these earlier findings, the current results are indeed surprising. Not only was no support found for the proposed hypothesis; the negative correlation between treatment dose and infarct size actually contradicted it. One explanation could be that our high dose capsules did not manage to keep the serum estrogen concentrations high enough during the entire study, since they were actually about half of the pellet induced serum concentrations on day 14, at the time of MCAo. Another explanation might be that a high estrogen dose is only one of several methodological parameters needed for the detrimental effect to occur, and that those other elements were currently lacking. Otherwise, the results presented here suggest that in the studies where estrogen treatment have caused enlarged infarcts, some other factor rather than high delivered dose has been the culprit. The fact that all studies reporting detrimental effects of estrogens have used the slow-release pellets^13^ remains hard to explain. It could be that some pellet component other than the active ingredient affects the ischemic stroke, but such a claim is merely speculative and perhaps far-fetched given that there are also examples of pellet studies demonstrating neuroprotection^17^,^18^.

This study did not include any native (non-ovariectomized) rats, but based on earlier studies, the 17β-estradiol concentrations in the low-dose group are within, or on some occasions slightly below, the physiological range^10^,^11^. However, no significant difference was found between the low-dose and vehicle groups on any blood sampling day. This was unexpected since the chosen low-dose regimen seemed suitable based on previous thorough administration studies from our laboratory^10^,^11^. This lack of difference may be attributed to uncertainties when measuring very low levels of serum 17β-estradiol. We have previously worked with radioimmunoassay (17b-estradiol double antibody, KE2D; Siemens Healthcare Diagnostics Inc., Tarrytown, NY, USA) for such analyses but since this kit is no longer available on the market an ELISA kit was used in the present study.

Coefficients of variation of the infarct size were higher than expected in the present study. In the comparison between groups, the p-value was just above the significance level and it could be that a difference would have been found with a lower outcome variation or larger groups. Several issues have been raised about the field of preclinical stroke research^19^ and variability in the extent of ischemic damage, resulting in low statistical power, is one of them^20^. A few studies have addressed this problem by studying how e.g. strain^21^,^22^, filament coating^23^,^24^ and filament insertion^25^ affect variation in infarct size; however, since the possible methodological variations are almost infinite it is hard to experimentally test all combinations to find the optimal model. In a meta-analysis from 2013 we extracted data from over 300 articles and analyzed method parameters’ impact on variability and found that the Wistar strain and silicone coated filaments were preferable in this aspect^26^. Although not a part of the original hypotheses of that study, Laser Doppler flowmetry usage also seemed to reduce variability. Previously, a study similar to the one described herein was conducted in our lab^15^ and the problem with large variability in that study prompted us to improve our model based on what the literature (including our meta-analysis) suggested. Among other things, we changed from Sprague Dawley to Wistar rats, introduced a Laser Doppler flowmetry system and, to increase power, occlusion time was lowered in order to reduce mortality and group sizes were doubled (n-value of the current study was approximately four times the average in similar preclinical stroke studies^26^). However, despite our efforts these modifications did not reduce variability. Laser Doppler flowmetry was in this study only used to confirm decrease in cerebral perfusion at filament insertion, and not to exclude animals. Exclusion of animals with insufficient blood flow decrease could theoretically reduce variability, but in the current study, the correlation between LDF decrease and infarct size was poor. In addition, there is a possible risk attached to the use of LDF for exclusion; if the effect of a substance is mediated through changes in cerebral blood flow, this may be masked. Although it is, for practical and ethical reasons, considered desirable with homogenous infarcts when working with animals, patients in the clinic present with a considerable heterogeneity in terms of stroke type, extent, duration of ischemia and severity^27^. This is worth mentioning since the ultimate goal of preclinical stroke research is to be able to translate the results to humans. Nevertheless, high variability requires large group sizes to maintain adequate statistical power. The recent initiative to perform animal studies as large multicenter studies with the same stringency as clinical trials could be a step in the right direction to overcome the problem with low statistical power and at the same time, through methodological differences between laboratories, introduce heterogeneity to further examine the robustness of results^28^.

Another possible weakness of the study is that the animals were allowed to survive only for 24 hours after induction of ischemia. It is known that the infarct changes also after this point in time^29^,^30^ and this could potentially hamper generalization of the findings. However, in previous studies on the effects of estrogens on ischemic stroke, infarct measurement after 24 h was the most common approach^14^ and it has been used both in studies where estrogens were neuroprotective^17^,^31^ and neurodamaging^7^,^8^. The limitations of 2, 3, 5-triphenyltetrazolium hydrochloride as a method for infarct measurement have to be considered as well, e.g. less clearly defined demarcation of infarct in white matter due to low density of mitochondria. The potential problem of infiltrating inflammatory cells containing intact mitochondria should not be an issue in the current study since the animals were sacrificed after 24 h^32^.

Infarct size was the only damage outcome in this study, except for the functional outcome of the modified sticky-tape test. Expansion of the scope of outcomes in future studies, e.g. regarding neuronal death, could add further information and thus provide a more elaborate description of the relation between estrogens and ischemic stroke.

An 8-point score based on recommendations from the Stroke Therapy Academic Industry Roundtable (STAIR)^33^, has previously been used to assess methodological quality in preclinical stroke studies^34^. Out of these eight criteria, six were met (randomization, monitoring of physiological parameters, assessment of dose-response relationship, masked outcome measurement, assessment of outcome days 1–3 and combined measurement of lesion volume and functional outcome). Assessment of optimal time window and assessment of outcome at days 7–30 were not fulfilled. Moreover, in the experimental design and during manuscript preparation, the authors adhered to the ARRIVE-guidelines (Animal Research: Reporting of *In Vivo* Experiments)^35^.

No support was found for the hypothesis that 17β-estradiol can be both neuroprotective and neurotoxic merely depending on dose. In fact on the contrary, the findings presented herein indicate that the higher the dose of 17β-estradiol, the smaller the infarct. If high estrogen doses/plasma concentrations *per se* cause increased stroke damage, such a phenomenon is not very robust, and seems to depend on very tight dose ranges and/or other experimental circumstances.

## Material and Methods

### Animals

A total of 130 female Wistar rats (Taconic Europe, Ry, Denmark; 14 weeks) were kept in 21 °C on a 12-hour light/dark cycle and with standard rodent chow and water provided *ad libitum*. Preoperatively the rats were housed two and two with access to nesting material, wooden chew sticks and cardboard cylinders and solitarily without enrichment postoperatively. The study was approved by the Local Ethics Committee for Animal Care and Use at Linköping University and all procedures were conducted in accordance with the National Committee for Animal Research in Sweden and Principles of Laboratory Animal Care (NIH publication no. 86–23, revised 1985).

### Experiment outline

One hundred and twenty rats were randomly allocated (Random Sequence Generator, random.org) into three groups that were ovariectomized and administered vehicle (vehicle group; n = 40), low-dose 17β-estradiol capsules (low-dose group; n = 40) or high-dose 17β-estradiol capsules (high-dose group; n = 40) on day 0. After two weeks (day 14) the rats were subjected to MCAo and on day 15 the rats were sacrificed and the damage was evaluated by the modified sticky-tape test and lesion measurement. Ten animals in each group were also blood sampled (day 2, 7 and 14) for 17β-estradiol concentration measurement. Another group of rats (pellet group; n = 10) were ovariectomized and administered high-dose 17β-estradiol slow-release pellets (day 0) and blood sampled (days 2, 7 and 14) to enable comparison of obtained 17β-estradiol levels between the capsules and the pellets. See Fig. 1 for an overview of the experiment.

### Blinding

One experimenter, blinded to treatment, performed all stroke surgeries and behavioral testing. Another experimenter performed ovariectomies, hormone administrations, blood samplings, sacrifices and infarct size analyses. Infarct size analyses were performed in a randomized and blinded fashion.

### Surgical procedures

#### Ovariectomy and administration of 17β-estradiol

Ovariectomies were performed via the dorsal route^36^. Subsequently, the rats were administered 17β-estradiol or vehicle in silastic capsules as described previously^36^. 30 mm segments of silastic tubing (Inner/outer diameter: 1.575/3.175 mm, Dow Corning, VWR International, Buffalo Grove, IL, USA) were filled with 17β-estradiol (Sigma-Aldrich Sweden AB, CAS# 50–28–2, Stockholm, Sweden) dissolved in sesame oil (Sigma-Aldrich Sweden AB, CAS#8008–74–0, Stockholm, Sweden) and capped with 5 mm pieces of wooden applicator sticks (Birch, length 15 cm, diameter 2 mm, SelefaTrade AB, Spånga, Sweden). The capsules were placed in two subcutaneous pockets dissected caudally in the loose skin of the rat’s neck, one on each side. All rats in the MCAo groups received two capsules; the vehicle group received two vehicle capsules (only sesame oil), the low dose group received one vehicle capsule and one 180 μg/mL capsule and the high dose group received two 50 000 μg/mL capsules. The doses were chosen based on earlier results from our lab; the low dose has previously been neuroprotective^37^ whereas the high-dose regimen has been shown to produce levels of 17β-estradiol higher than or equivalent to the neurotoxic slow-release pellets (unpublished pilot study). The rats in the pellet group were subjected to the same procedures, except that instead of two silastic capsules, one slow-release pellet (90-day release, 1.5 mg, Innovative Research of America, Sarasota, FL, USA) was inserted.

#### MCAo

Left-sided, 30 minute, intraluminal filament MCAo was performed based on the descriptions by Koizumi^38^ and Longa^39^. A 2 cm cervical midline incision was made and the common (CCA), internal (ICA) and external (ECA) carotid arteries were freed from surrounding tissue. After ligation of CCA and ECA, the ICA was temporarily clipped (8 mm artery clip, Rebstock Instruments Gmbh, Dürbheim, Germany). Thereafter, a 30 mm silicone coated 4–0 nylon suture (403756, Doccol, Redlands, CA, USA) was inserted in CCA and advanced up ICA approximately 18–20 mm. Laser Doppler flowmetry (LDF; moorVMS-LDF, Moor Instruments, Axminster, Devon, UK) of left hemisphere perfusion was used to confirm correct filament placement with probe placed against the skull through the temporal muscle^40^. After 30 minutes of occlusion (rat kept anesthetized), the filament was withdrawn and ICA was permanently ligated. Postoperatively, the rats were allowed to recover in a heated cage (30 °C) for 15 minutes and to facilitate eating, water-soaked food pellets were provided in a petri dish on the cage floor.

#### Anesthesia, analgesia and physiological monitoring

The rats were anesthetized with Isoflurane (Forene^®^, Abbott Scandinavia AB, Solna, Sweden), 4.5% for induction and 1.5% for maintenance delivered in a 30/70 mixture of O_2_/N_2_O, for ovariectomy, blood sampling and MCAo. Carpofen (462986, Rimadyl Vet, Pfizer ApS, Ballerup, Denmark) 5 mg/kg body weight subcutaneously was used for ovariectomy pain relief. Before MCAo, animals were administered 1.25 mg/kg bodyweight bupivacaine (Marcain, AstraZeneca, Södertälje, Sweden) subcutaneously in addition to flavored paracetamol (Paracetamol Apofri, Apofri AB, Danderyd, Sweden) provided at a concentration of 1 mg/mL in the drinking water, two days before MCAo for habituation and until sacrifice. Before MCAo, 5 mL saline was given as fluid replenishment. Saturation, respiratory rate and heart rate were recorded by pulse oximetry (SLS-MO-00404, MouseOx, Allison Park, PA, USA), these parameters are presented in Table 3.

### Measurement procedures

#### Blood sampling and 17β-estradiol immunoassay

Blood sampling was performed by venipuncture of the hind leg and the serum samples were analyzed in duplicate using enzyme-linked immunosorbent assay (Mouse/Rat Estradiol ELISA kit, Calbiotech, Spring Valley, CA, USA). This kit previously performed best overall compared to several other immunoassay kits^41^. Samples expected to have concentrations above the range of the kit were diluted 1:16 according to recommendation from the kit manufacturer (phosphate-buffered saline with 1% bovine serum albumin; Sigma-Aldrich Sweden AB, CAS#9048–46–8, Stockholm, Sweden).

#### Body weight

Body weights of the MCAo groups were recorded on days 0, 2, 7, 14 (pre-MCAo) and 15 using the same 10 animals per group that were blood sampled. For the pellet group, weights were recorded for all animals on days 0, 2, 7 and 14.

#### Modified sticky-tape test

The modified sticky-tape test (MST) was performed similarly to the procedure Sughrue *et al.* described^42^. Briefly, a 30 mm segment of autoclave tape was wrapped around the right forepaw (impaired side; contralateral to the ischemic hemisphere). The rat was then placed in a transparent cage (36 × 19.5 × 18.5 cm) and during 30 seconds, time spent on attempting to remove the tape sleeve either by biting or scratching with the left paw, was recorded. On the few occasions when the rat managed to remove the tape sleeve, the time attending to the stimulus was set to the maximum 30 seconds. The test was performed pre-MCAo and 24 hours post-MCAo and the average of two testing sessions per rat on each occasion was used.

#### Lesion measurement

Under anesthesia the rat was decapitated using a rodent guillotine, the brain was dissected out and cooled in ice water for 5 minutes and sliced in 2 mm slices using a rat brain matrix (RBM-4000, ASI Instrument Inc., USA). Subsequently, the slices were soaked in 2% 2, 3, 5-triphenyltetrazolium hydrochloride solution (TTC; Sigma-Aldrich Sweden AB, CAS# 298–96–4, Stockholm, Sweden) at 37 °C for 15 minutes and scanned (ScanJet 2c, Hewlett-Packard, Palo Alto, CA, USA). Infarcts were measured in a similar way as described by Goldlust *et al.*^43^, with an automatic 40% green spectrum threshold (SigmaScan Pro 5, Systat Software Inc, San Jose, CA, USA). Edema correction was performed according to the following formula:

Corrected infarct volume, as part of one hemisphere = [crude infarct area × [contralateral hemisphere/ipsilateral hemisphere]]/contralateral hemisphere

### Exclusion criteria

Rats that died before the end of MCAo surgery (excluded mortality) were excluded from the study. In contrast, rats that died after MCAo (included mortality) were included in the subsequent non-parametric analyses, assigned worst possible outcome (see Statistics and Table 1).

### Statistics

With an increase in lesion size of 40% in the high dose group, a CV% of 50 for infarct sizes and sample sizes of 40, the statistical power would be 0.84. To account for differences in mortality between the groups, a non-parametric “total outcome” Kruskal-Wallis model with Dunn’s post-hoc test was used for comparisons between groups regarding infarct size and behavioral testing (MST). Rats that died after MCAo (included mortality; see Table 1) were included in the analysis and were assigned the worst possible outcome. As a complementary analysis, after it had been observed that the relation between dose and outcome seemed linear, Spearman correlation was performed. For 17β-estradiol concentrations, three Kruskal-Wallis tests with Dunn’s post hoc test were used to compare groups on day 2, 7 and 14. Non-parametric analysis was chosen because a few concentration outliers would have had too large impact in a parametric analysis. Mixed-design (split-plot) ANOVA was used to compare body weights over time between groups, with measuring day as within-subject factor and group as between-subject factor. Weights were normalized to each animal’s value pre-ovariectomy (day 0). In all analyes, p values below 0.05 were considered significant.

## Additional Information

**How to cite this article**: Ingberg, E. *et al.* Effects of high and low 17β-estradiol doses on focal cerebral ischemia in rats. *Sci. Rep.* **6**, 20228; doi: 10.1038/srep20228 (2016).

## Figures and Tables

**Table 1:** Included mortality (post-MCAo).

Group	Day 14	Day 15
Vehicle	2	1
Low-dose	2	2
High-dose	0	0

**Table 2:** Serum 17β-estradiol concentrations.

Day	Median [pg/mL]	Interquartile range [pg/mL]
Vehicle capsule group		
2	4.7	3.1–6.3
7	4.3	2.6–8.5
14	3.6	2.6–6.9
Low-dose capsule group		
2	8.3	8–13.1
7	4.3	3.1–6.8
14	3.9	2.1–13.3
High-dose capsule group		
2	3581.2	2402.6–4674.2
7	562	328.2–877.4
14	245.6	153–279
Pellet group		
2	381.6	348.8–428.8
7	430.4	317.4–635
14	427.2	415.4–439.6

**Table 3:** Physiological parameters during surgery.

Group	Average oxygen saturation, mean ± SEM [%]	Average heart rate, mean ± SEM [bpm]	Average respiratory rate, mean ± SEM [brpm]
Vehicle group	96.4 ± 0.65	407.5 ± 11.9	52.0 ± 2.2
Low-dose group	95.8 ± 0.76	429.1 ± 10.3	55.1 ± 2.0
High-dose group	96.5 ± 0.69	383.9 ± 8.7	54.1 ± 1.8
